# Whole genome sequence and comparative genomics analysis of multidrug-resistant *Staphylococcus xylosus* NM36 isolated from a cow with mastitis in Basrah city

**DOI:** 10.1186/s43141-023-00606-6

**Published:** 2023-12-07

**Authors:** Hassan M. Al-Tameemi, Husam Al-Hraishawi, Murtakab Y. Al-Hejjaj, Noor S. Abdulah, Haider R. Alrafas, Yessar A. Dawood

**Affiliations:** 1 Microbiology Department, College of Veterinary Medicine, Basrah University, Basrah, 61004 Iraq; 2 Physiology Department, College of Medicine, Misan University, Amarah, Misan Iraq; 3 Pharmacognosy and Medicinal Plants Department, College of Pharmacy, Basrah University, Basrah, Iraq

**Keywords:** *Staphylococcus xylosus*, Whole genome sequencing, Mastitis, Biofilm, Antibiotic resistance

## Abstract

**Background:**

*Staphylococcus xylosus* is a coagulase-negative, gram-positive coccus that is found in the environment and as a commensal organism on the skin and mucosal surfaces of animals. Despite the fact that *S. xylosus* is considered a nonpathogenic bacterium, several studies have linked *S. xylosus* to opportunistic infections in both animals and humans. During an investigation of mastitis-causing agents in the governorate of Basrah, Iraq, we identified an antibiotic-resistant strain of *S. xylosus* NM36 from a milk sample from a cow with chronic mastitis. In addition to robust biofilm formation, multiple antibiotic resistance phenotypes were found. To further understand the genetic background for these phenotypes, the full genome of *S. xylosus* NM36 was analyzed.

**Results:**

The genome consisted of a single circular 2,668,086 base pairs chromosome containing 32.8% G + C. There were 2454 protein-coding sequences, 4 ribosomal RNA (rRNA) genes, and 50 transfer RNA (tRNA) genes in the genome. In addition, genetic variation was studied by searching sequence data against a representative reference genome. Consequently, single-nucleotide polymorphism analysis was conducted and showed that there were 46,610 single-nucleotide polymorphisms (SNPs), 523 insertions, and 551 deletions. In order to overcome antibiotics, *S. xylosus* NM36 had been armed with several antibiotic resistance genes from several groups and families. The genome annotation service in PathoSystems Resource Integration Center (PATRIC) and Rapid Annotation using Subsystem Technology (RAST) annotation servers showed that there are multiple antimicrobial resistance elements, including antibiotic inactivation enzymes (BlaZ family, FosB), antibiotic resistance gene clusters (TcaB, TcaB2, TcaR), proteins involved in methicillin resistance (LytH, FmtA, FemC, HmrB, HmrA), TetR family transcriptional regulators, and efflux pumps conferring antibiotic resistance (NorA). In addition, we investigated and categorized the biofilm and quorum-sensing elements of the NM36 strain and found that it has multiple subsets of biofilm regulators, confirming its pathogenic nature.

**Conclusions:**

These findings necessitate a reevaluation of microbial and clinical interventions when dealing with coagulase-negative staphylococci, particularly in the context of studies pertaining to public health. This is the first time, to our knowledge, that the entire genome of *S. xylosus* has been sequenced in Iraq.

**Supplementary Information:**

The online version contains supplementary material available at 10.1186/s43141-023-00606-6.

## Background

*Staphylococcus xylosus* are coagulase-negative, gram-positive cocci that are widespread in the environment and are commensal on the skin and mucosal surfaces of animals. Despite the fact that *S. xylosus* is considered a nonpathogenic bacterium, opportunistic infections in animals and humans have been linked to *S. xylosus* in several studies. *S. xylosus* is widespread and could be found in a variety of environments, including contaminated water, meat, fodder, and soil surfaces [4, 15, 19, 27, 29, 31, 34, 36]. It has been shown that coagulase-negative staphylococci (CoNS) can play an important role in bovine intramammary infections and also share mobile genetic elements that carry virulent factors such as antibiotic resistance markers with other family members, including *S. aureus* [21, 34, 62]. In addition to its clinical relevance, S. *xylosus* may contribute to the pathogenicity of other staphylococci through horizontal gene transfer of antibiotic resistance elements such as the SCC*mec* type 11 region and tetracycline resistance [33, 37]. Since *S. xylosus* is becoming increasingly infectious alongside other staphylococci, it is essential to investigate the genome of this ubiquitous commensal. *S. xylosus* has been sequenced at the genome level far less frequently than *S. aureus* in the public domain, and none has been performed in Iraq. Even though previous studies have done genome annotation and analysis of *S. xylosus*, a thorough exploration of the pathogenicity of this bacterium based on genomic information gained through next-generation sequencing (NGS) is still necessary, particularly for relating data from various geographic regions [29]. During an investigation of mastitis-causing agents in the governorate of Basrah, Iraq, we identified an antibiotic-resistant strain of *S. xylosus* (coagulase-negative staphylococci (CoNS) from a milk sample of a cow with chronic mastitis. Using disc diffusion method, we found that this isolate was resistant to methicillin, ampicillin, cefoxitin, oxacillin, and tetracycline but was sensitive to vancomycin. In addition, the strain showed notable biofilm-formation capacity. To further understand the genetic background for these phenotypes, *Staphylococcus xylosus* NM36 whole genome sequencing was undertaken to find variant information and to perform gene annotation on key genes relevant to antibiotics and biofilm formation.

## Methods

*S. xylosus* NM36 was isolated from a clinical mastitis of a cow. Isolation and identification were conducted using standard microbiological procedures. Confirmation was achieved by sequencing the PCR product of the 16 s RNA using universal primers 27F and 1492R [22] and blast analysis using the NCBI database [53]. Genomic DNA was extracted using the QIAamp DNA Mini Kit, Qiagen USA, catalog number 51304, according to the manufacturer’s instructions. DNA samples were sent for whole genome sequencing using the Illumina platform sequencer (Macrogen, Korea). After conducting quality control (QC), samples for library construction were subjected to random DNA fragmentation, followed by 5′ and 3′ adapter ligation. Adapter-ligated fragments were amplified and purified by PCR and gel. The library was fed into a flow cell for cluster generation, where fragments were captured on a lawn of surface-bound oligos that were complementary to the library adapters. By means of bridge amplification, each fragment was amplified into separate clonal clusters. After the generation of clusters, the templates were ready for sequencing. Following sequencing, raw reads were analyzed for overall read quality, total bases, total reads, and GC (%), and basic statistics were calculated. In order to reduce biases in analysis, FastQC [3] and quality-filtering processes were performed. The quality of the produced data was determined by applying the phred quality score at each cycle Q20 (%) and Q30 (%) which helps measure the quality of the identification of the nucleobases generated by automated DNA sequencing [20]. The raw reads were de novo assembled into contigs using the SPAdes v.3.5 bioinformatics tool [6].

### Genome analysis and comparison with other genomes

*Staphylococcus xylosus* NM36’s assembled genome was submitted to PATRIC’s comprehensive genome analysis service, which uses PATRIC’s curated collection of representative antimicrobial resistance (AMR) gene sequence variants [63]. In order to map reads obtained from sequencing, *Staphylococcus xylosus* was used as a reference genome. Filtered reads were mapped to the reference genome with BWA—Burrows-Wheeler Aligner [35]. After read mapping, Picard and SAMTools were used to remove duplicate reads and find variant information [47]. Broad Institute, [16]. To contrast the mapping results, the assembled genome was further annotated for functional genes in subsystem categories using the classic RAST and RASTtk server [5, 11] and the SEED tool [45]. In all annotation and comparison processes, the similarity threshold was at least 95% identity. The annotated features were further verified and illustrated using the PROKSEE server [25] which was used for identifying conserved and unique sequence features and to generate high-quality maps as previously described [56]. Using the SEED tool, the *Staphylococcus xylosus* NM36 genome was further compared to other closely related genomes (ANMR00000000.1, CP007208.1, CP008724.1, CP031275.1, CP066721.1).

### Genome submissions to NCBI GenBank

The genome sequence of *Staphylococcus xylosus* NM36 has been deposited at GenBank—DDBJ/ENA/GenBank under the accession number GenBank JARUHN000000000.1. The annotation was added by the NCBI Prokaryotic Genome Annotation Pipeline (PGAP) [57].

### SNP and INDEL discovery

The mapped data were examined for single-nucleotide polymorphism (SNPs) and insertion/deletion (INDEL) variations compared to the reference genome. In this analysis, the reference genome is based on RefSeq assembly accession: GCF_000709415.1 (CP008724.1). After removing duplication and finding variants’ information with SAMTools, each variant’s information was gathered and classified by chromosomes or scaffolds.

### Phylogenetic analysis

The phylogenic analysis package at PATRIC [63] was used to categorize reference and representative genomes. PATRIC incorporated the reference and representative genomes into the phylogenetic analysis included in the report on Comprehensive Genome Analysis. In summary, Mash/MinHash identified the closest reference and representative genomes. From these genomes, PATRIC global protein families (PGFams) were selected to ascertain the phylogenetic placement of this genome. The nucleotides of these sequences were mapped, and multiple sequence comparison by log expectation (MUSCLE) was used to align the protein sequences of these families. The combined set of amino acid and nucleotide alignments was concatenated into a data matrix, and RaxML was used to analyze this matrix, with rapid bootstrapping used to generate the support values in the tree. In addition, a phylogenetic tree was built based on the 16 s RNA sequence relationship using the NCBI Tree Viewer (TV). For this comparison, we selected only the sequences whose genomes had been fully sequenced and deposited in the NCBI database.

## Results

### Genome annotation

**Fig. 1:**
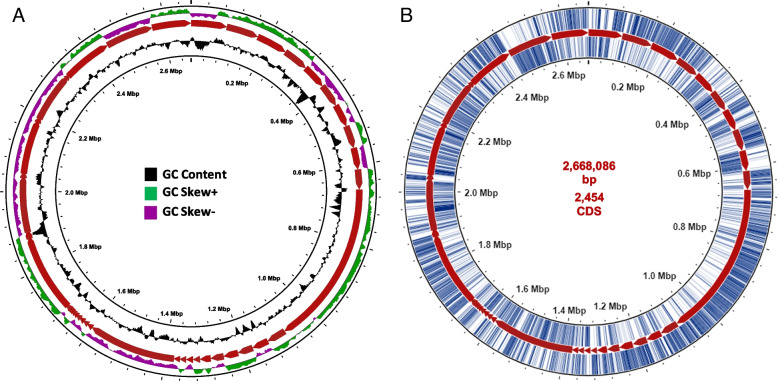
Circular genome representation of *Staphylococcus xylosus* NM36 (PROKSEE server). The inner most ring represents chromosome position, and the red-colored ring represents the genome backbone (in contigs). **A** GC content (black) and GC skew (green represents values greater than the genome average, whereas purple represents value less than the genome average. **B** The open-reading frames on the forward strand (outside of the backbone) and reverse strands (inside of the backbone)

**Fig. 2:**
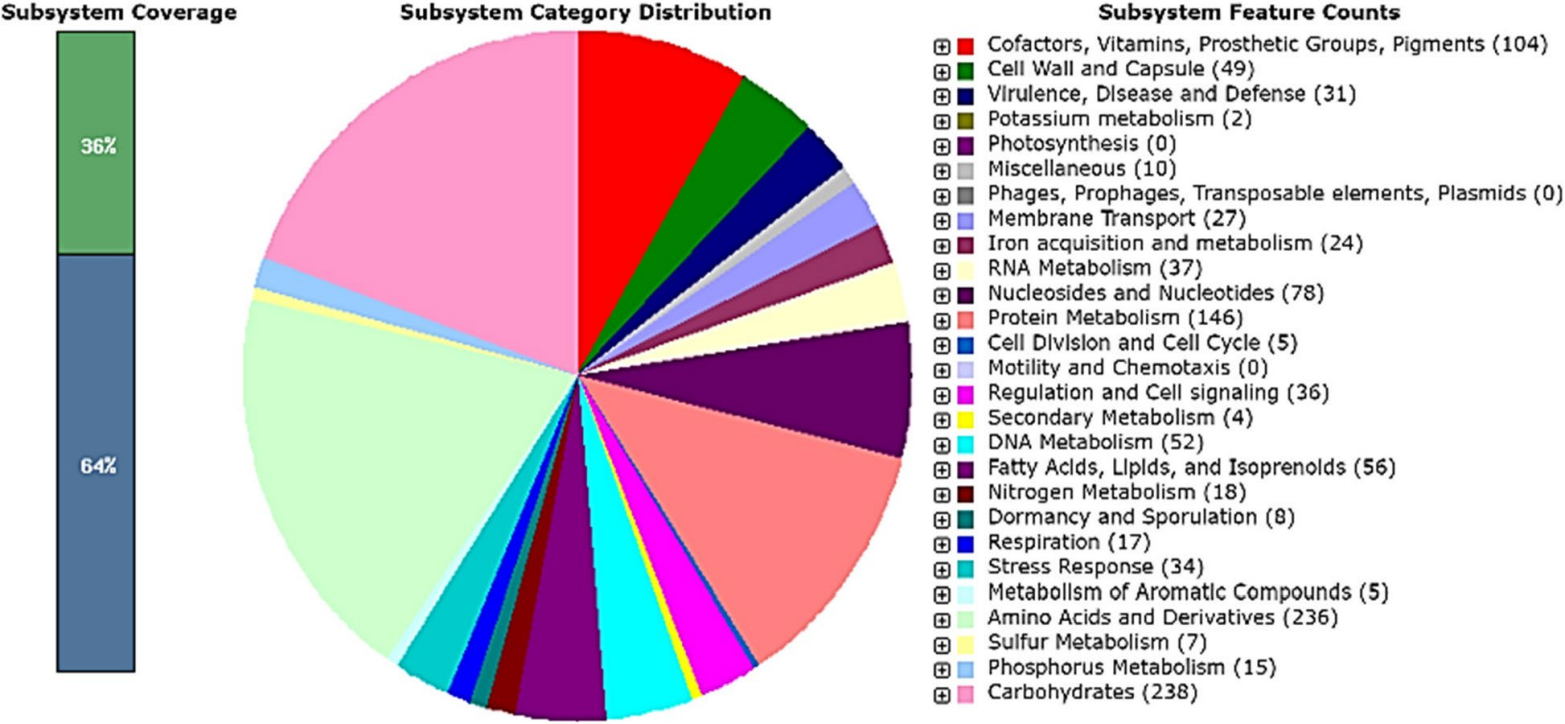
*Staphylococcus xylosus* NM36 distribution statistics for subsystem categories. Using the Rapid Annotation System Technology (RAST) server, the genome was annotated. The pie chart displayed the number of each subsystem feature, and the SEED viewer displayed the subsystem coverage. The green bar of the subsystem coverage represents the proportion of proteins included in the subsystems, while the blue bar represents the proportion of proteins excluded from the subsystems

Based on the annotation data and the contrast to other genomes in PATRIC within the same species, this genome is considered of good quality. This was confirmed by the phred quality score of bases over Q20 and Q30 which were 98.47% and 94.34%, respectively, after read filtering. The Comprehensive Genome Analysis showed that this assembled genome has 73 contigs, with a total length of 2,668,086 bp, 2454 coding proteins (Table 1, Additional file 1: Appendices A: Table S1). Furthermore, the average GC content is 32.8%. A schematic representation for GC content and GC skew analysis is shown in Fig. 1. A subsystem is a set of proteins that together implement a specific biological process or structural complex. The annotation process included an analysis of the subsystems unique to this genome, which revealed that there are 278 subsystems. An overview of the subsystems for this genome is provided in Fig. 2. Single-nucleotide polymorphism (SNP) analysis showed that there were 46,610 SNPs, 523 insertions, and 551 deletions compared to the reference genome (Additional file 1: Appendices B: Table S2).

**Table 1:** Summary for the genome analysis report of NCBI prokaryotic genome annotation pipeline (PGAP)

**Genome annotation pipeline (PGAP)**	**Results**
Total length	2,668,086 bp
GC content %	32.8
Number of contigs	73
Number of subsystems	278
Genes (total)	2530
CDSs (total)	2469
Genes (coding) proteins	2454
CDSs (with protein)	2454
RNA genes	61
rRNAs	2, 2, 3 (5S, 16S, 23S)
tRNAs	50
Pseudo genes (total)	15
CDSs (without protein)	15
Contig L50	7
Contig N50	126,244
Plasmids	0

### Phylogenetic analysis

**Fig. 3:**
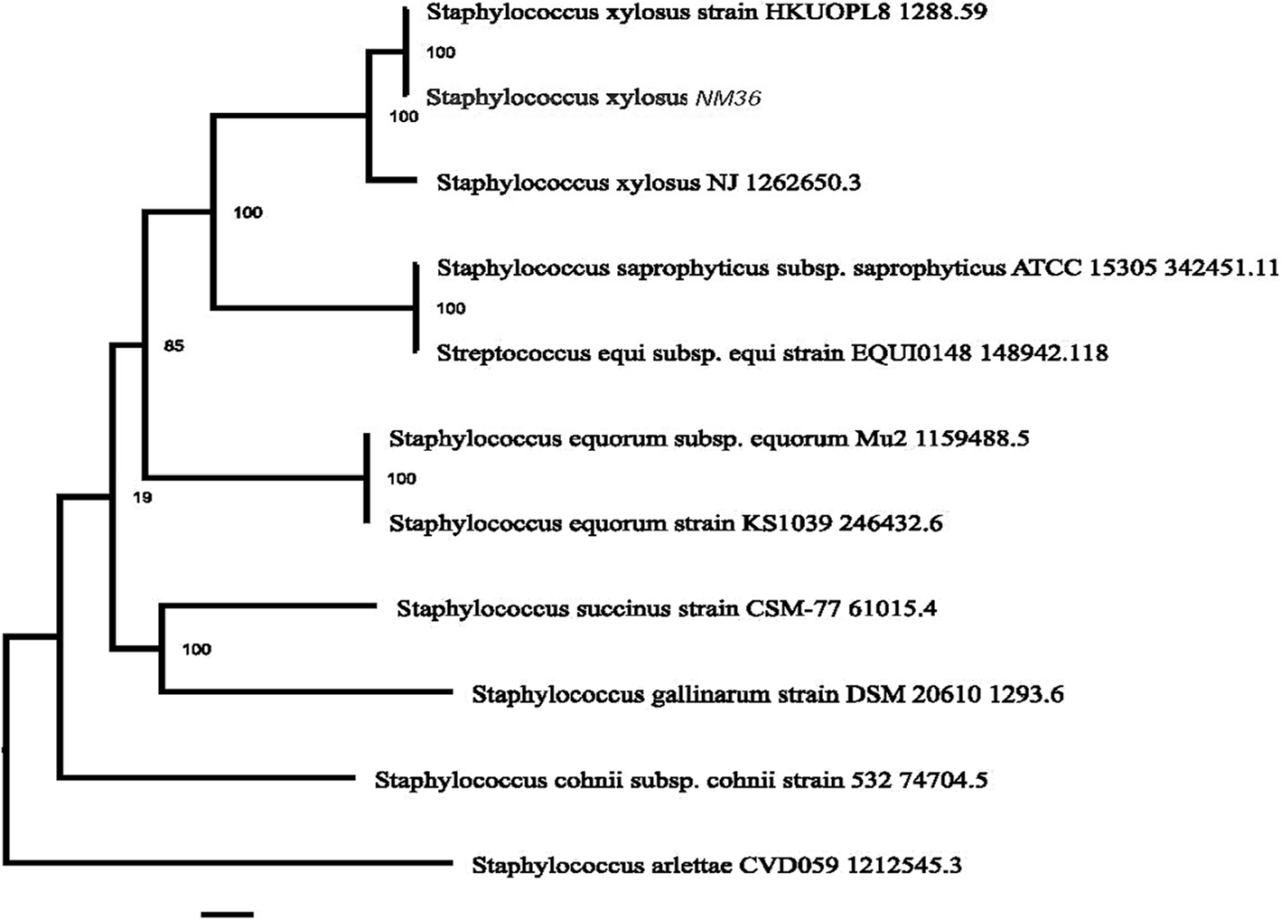
Phylogenic relationship representation of *S. xylosus* NM36 based on the genome features (using the genome annotation service in PATRIC)

**Fig. 4:**
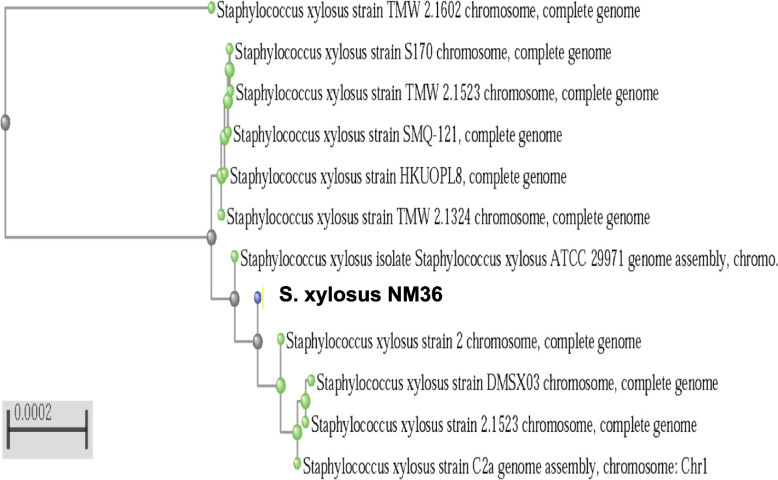
16S rRNA sequences distance tree depicting the relationship between *S. xylosus* NM36 (shown in yellow) and related *S. xylosus* strains in the NCBI database (strains with complete genome) using NCBI TV

**Fig. 5:**
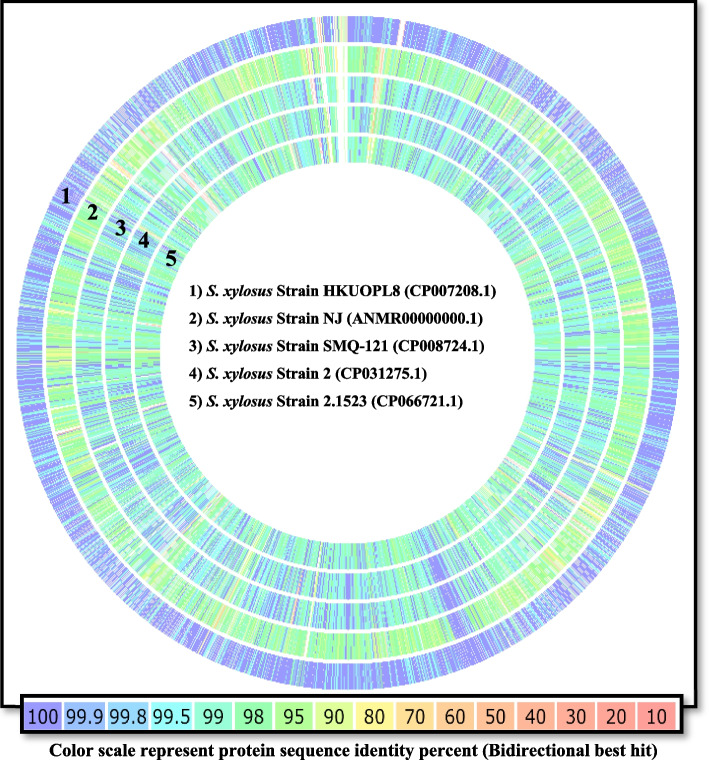
Graphical genome comparison map of the NM36 strain (reference) with five closely related species using the Seed Viewer sequence-based comparison tool in the RAST server. From outside to inside rings: (1) strain HKUOPL8 (CP007208.1), (2) strain NJ (ANMR00000000.1), (3) strain SMQ-121 (CP008724.1), (4) strain 2 (CP031275.1), and (5) strain 2.1523 (CP066721.1). From purple (100%) to pale red (10%), the colors represent the similarity of amino acids to the reference genome. The NM36 reference strain’s genome is not depicted in the figure

PATRIC provided the reference and representative genomes, which were included in the phylogenetic analysis. The reference and representative genotypes closest to them were determined and illustrated in Fig. 3. It turned out that strains *S. xylosus* HKUOPL8 and NJ (ANMR00000000.1) have the highest similarity. In addition, a phylogenetic tree was built based on the 16 s RNA sequence relationship using NCBI Tree Viewer (TV) for the sequences for which their genome had been fully sequenced (Fig. 4). This allowed us to select five related genomes for sequence-based comparison by the RAST tool, which revealed that strain NM36 and the other closely related strains possess a high abundance of coding DNA sequences (CDS), mainly coding for carbohydrate and amino acid metabolism (Figs. 2 and 5). In all 5 genomes, there are 1188 genes with the same annotated functions with at least 95–100% identity.

### Resistance to antibiotics and toxic compounds

A significant number of the genes annotated have homology to known virulence factors, transporters, drug targets, and antimicrobial resistance genes. To filter the results, we combined and filtered the results from the SEED, PATRIC, and PROKSEE servers into one list. Then we investigated AMR gene sequence variants and assigned to each AMR gene a functional annotation, a drug class, and the specific antibiotic it confers resistance to. A summary of the AMR genes annotated in this genome and the corresponding AMR mechanisms is provided in Table 2.

**Table 2:** * S. xylosus* NM36 genome analysis for antibiotics resistance. The annotation was based on the protein domain database [38–41]

#	Contig	Start	Stop	Strand	Length (bp)	Function
1.	JARUHN010000001.1	83,305	84,474	+	1170	Multidrug resistance protein B-efflux pumps. MFS family multiresistance [17, 46]
2.	JARUHN010000001.1	223,144	224,019	+	876	LytH protein, involved in methicillin resistance/N-acetylmuramoyl-L-alanine amidase (EC 3.5.1.28) domain [23]
3.	JARUHN010000002.1	27,376	25,487	-	1890	Membrane component of multidrug resistance system—conserved domains: drug resistance transporter, EmrB/QacA subfamily; SmvA family efflux MFS transporter [17]
4.	JARUHN010000002.1	28,165	28,713	+	549	TetR family regulatory protein of MDR cluster. Multidrug resistance, 2-protein version found in gram-positive bacteria [38]
5.	JARUHN010000002.1	65,241	65,846	+	606	Transcriptional regulator, TetR family
6.	JARUHN010000002.1	137,322	136,774	-	549	
7.	JARUHN010000004.1	102,889	103,473	+	585	
8.	JARUHN010000005.1	115,390	114,833	-	558	
9.	JARUHN010000024.1	3420	3983	+	564	TetR family regulatory protein
10.	JARUHN010000027.1	12,167	12,718	+	552	
11.	JARUHN010000002.1	30,005	28,797	-	1209	Teicoplanin resistance, associated membrane protein TcaB [10]
12.	JARUHN010000002.1	31,832	30,447	-	1386	Teicoplanin resistance, associated membrane protein TcaA [10]
13.	JARUHN010000002.1	32,591	32,127	-	465	Teicoplanin resistance, associated HTH-type transcriptional regulator TcaR, teicoplanin resistance in *Staphylococcus* [10]
14.	JARUHN010000002.1	111,486	112,688	+	1203	Bicyclomycin resistance protein, TcaB2 [10]
15.	JARUHN010000002.1	142,698	141,520	-	1179	Chloramphenicol resistance protein
16.	JARUHN010000003.1	100,532	101,692	+	1161	Quinolone resistance protein, *norA* [18]
17.	JARUHN010000006.1	57,874	54,734	-	3141	RND multidrug efflux transporter; Acriflavin resistance protein [44]
18.	JARUHN010000007.1	27,204	28,379	+	1176	FmtA protein, involved in methicillin resistance [65]
19.	JARUHN010000008.1	5175	4807	-	369	FemC, factor involved in methicillin resistance/glutamine synthetase repressor [26]
20.	JARUHN010000008.1	106,860	106,627	-	234	Acyl carrier protein/HmrB protein involved in methicillin resistance [30]
21.	JARUHN010000012.1	19,897	21,081	+	1185	HmrA protein, involved in methicillin resistance/amidohydrolase of M40 family [30]
22.	JARUHN010000022.1	24,055	26,580	+	2526	FmtC (MrpF) protein, involved in methicillin resistance/L-lysine modification of phosphatidylglycerol [65]
23.	JARUHN010000036.1	2573	2992	+	420	Fosfomycin resistance protein, FosB-BlaZ family, FosB [58]

### Quorum sensing and biofilm formation

Since the biofilm is essential to staphylococcal biology, several regulatory systems that take into account the physiological state of the cell, environmental cues, and the dynamics within the staphylococcal community tightly regulate the formation and disassembly of biofilms. A list for the annotated genes that are involved in biofilm formation in *S. xylosus* NM36 is listed in Table 3.

**Table 3:** *S. xylosus* NM36 genome analysis for genes involved in regulation and cell signaling—quorum sensing and biofilm formation. The annotation was based on the protein domain database [38–41]

#	Contig	Start	Stop	Strand	Length (bp)	Function
1.	JARUHN010000002.1	32,591	32,127	-	465	Transcriptional regulator TcaR (transcriptional inhibitors of the *ica* locus) [28]
2.	JARUHN010000003.1	71,220	73,370	+	2151	Transcriptional regulator of biofilm formation (AraC/XylS family) [24]
3.	JARUHN010000004.1	40,722	39,415	-	1308	Putative bifunctional autolysin Atl [9]
4.	JARUHN010000007.1	17,600	18,022	+	423	Putative Atl autolysin transcription regulator [9]
5.	JARUHN010000007.1	22,546	18,119	-	4428	Bifunctional autolysin Atl/N-acetylmuramoyl-L-alanine amidase (EC 3.5.1.28)/endo-beta-N-acetylglucosaminidase (EC 3.2.1.96) [9]
6.	JARUHN010000003.1	24,247	23,873	-	375	Staphylococcal accessory regulator A (SarA) [7]
7.	JARUHN010000021.1	2983	2300	-	684	Response regulator SaeR (*Staphylococcus* exoprotein expression protein R) [42]
8.	JARUHN010000012.1	75,289	76,059	+	771	RNA polymerase sigma factor SigB [32]
9.	JARUHN010000021.1	14,393	15,622	+	1230	Polysaccharide intercellular adhesin (PIA) biosynthesis N-glycosyltransferase IcaA (EC 2.4.-.-) [60]
		15,891	16,742	+	852	Polysaccharide intercellular adhesin (PIA) biosynthesis deacetylase IcaB [60]
		16,735	17,802	+	1068	Polysaccharide intercellular adhesin (PIA) biosynthesis protein IcaC [60]
		18,351	18,926	+	576	Biofilm operon ica ABCD HTH-type negative transcriptional regulator IcaR [60]
10.	JARUHN010000001.1	352,989	353,711	+	723	Staphylococcal respiratory response protein SrrA [43, 59]
		353,680	355,473	+	1794	Staphylococcal respiratory transmembrane histidine kinase protein SrrB [43, 59]
11.	JARUHN010000009.1	14,624	13,908	-	717	*agr*—accessory gene regulator A (response regulator) [8]
		15,932	14,637	-	1296	*agr*—accessory gene regulator C (sensor histidine kinase) [8]
		16,667	16,098	-	570	*agr*—accessory gene regulator B [8]
12.	JARUHN010000008.1	75,944	75,171	-	774	GTP-sensing transcriptional pleiotropic repressor CodY [49]
13.	JARUHN010000002	125,802	127,553	+	1752	Autolysis histidine kinase LytS [55]
		127,556	128,320	+	765	Autolysis response regulator LytR [55]
14.	JARUHN010000003.1	94,251	93,808	-	444	Transcriptional regulator MgrA (regulator of autolytic activity) [61]
15.	JARUHN010000001.1	414,891	415,553	+	663	Putative response regulator ArlR
		415,550	416,905	+	1356	Two-component system histidine kinase ArlS

## Discussion

Despite the fact that *S. xylosus* is considered a nonpathogenic bacterium, several studies have linked it to opportunistic infections in animals and humans. *S. xylosus* is widespread and can be found in numerous environments, such as contaminated water, animal feed, and soil surfaces [1, 2, 13, 14, 19, 27, 33, 36, 37, 62]. *S. xylosus* may contribute to the pathogenicity of other staphylococci via horizontal gene transfer of antibiotic resistance elements, such as the SCC*mec* type 11 region [37] or tetracycline gene transfer in *Staphylococcus xylosus* in situ during sausage fermentation, thereby exacerbating the risk of antibiotic resistance and posing a significant risk to public health [33]. In Iraq, there is limited information about *Staphylococcus xylosus*, which is reported occasionally during clinical investigations [2, 51] and similarly to other coagulase-negative staphylococci, and *S. xylosus* receives less interest compared to the more focus on *S. aureus*. The pathogenicity of staphylococci has been primarily linked to their capacity to resist antimicrobials and form biofilms. The initial attachment of bacteria to biotic and abiotic surfaces results in the accumulation of multilayered cell aggregates that constitute biofilm formation. This facilitates the internalization and survival of staphylococci within the host cells [54]. Therefore, strains that facilitate this trait are regarded as more virulent. *S. xylosus* NM36 possesses a number of virulence determinants that have been associated with the ability of staphylococci to adhere to biotic and abiotic surfaces, as well as the different phases of biofilm formation and antimicrobial resistance summarized in Tables 2 and 3. These results validate the initial phenotypes of multiresistance and biofilm formation observed during the initial isolation. Comparing the NM36 genome of *S. xylosus* to clinical reference strains revealed its arsenal of antibiotic resistance and virulence genes. In addition, *S. xylosus* NM36 contains 9 antibiotic resistance determinants responsible for resistance to 10 known antibiotics, including quinolone, methicillin, teicoplanin, bicyclomycin, chloramphenicol, fosfomycin, ampicillin, cefoxitin, oxacillin, and tetracycline. The NM36 genome harbors the *ica* operon and transcriptional regulator TcaR, both of which have been implicated in biofilm formation in staphylococci. It also contained the global regulators *agr* (accessory gene regulator), the main autolysin gene *atl* (autolysin), *sarA* (staphylococcal accessory regulator), and the two-component system *arlRS* and *srrAB*, which are involved in the regulation of adhesion and biofilm formation. Strain HKUOPL8 (CP007208.1) shares the maximum degree of protein similarity with NM36 (Figs. 3 and 5) [36]. According to the genome data on the NCBI website, strain HKUOPL8 (CP007208.1) was isolated from a clinical case (panda feces) [36], strain NJ (ANMR00000000.1) from a nasal swap (human), strain SMQ-121 (CP008724.1) from fermented sausage [31], strain 2 (CP031275.1) from a milker’s hand, and strain 2.1523 (CP066721.1) from fermented sausage. This variation in genome similarity may be attributable to lifestyle and isolation source differences, which may have affected the genetic composition of these isolates [48, 64]. INDELS are a significant source of genetic diversity that can significantly affect the properties or evolvability of a protein [52]. Single-nucleotide polymorphism (SNP) analysis showed that there were 46,610 SNPs, 523 insertions, and 551 deletions compared to the reference genome. Whether some of these mutations are advantageous, guiding the protein onwards towards a point of high fitness to current selective pressures, or not, will require additional research in the future. Similar to conventional molecular typing, it is probable that the genomes of isolates recovered from an outbreak or cluster of infections are closely related and may share pathogenic traits due to horizontal gene transfer [12]. In recent years, the widespread availability of whole genome sequencing (WGS) has made it possible to examine in greater detail patterns of spread, including the detection of previously undocumented transmission. Whole genome sequencing (WGS) can be used to investigate infectious disease epidemics and track the spread of infection, but unlike conventional molecular typing techniques such as *spa* typing, pulse-field gel electrophoresis (PFGE), and multilocus-sequence typing (MLST), WGS enables the comparison of entire genomes, thereby enhancing the resolution and accuracy of metabolic and subsystem maps [50]. However, the accumulation of genome sequences in the databases has been sporadic, with biased sampling of natural variation motivated primarily by medical and epidemiological priorities. For instance, sequencing epidemic lineages of methicillin-resistant *Staphylococcus aureus* (MRSA) is favored over sequencing sensitive isolates (methicillin-sensitive *S. aureus*: MSSA). As more diverse genomes are sequenced, a picture of a highly subdivided species with a limited number of relatively clonal groups (complexes) that dominate in specific geographic regions at any given time emerges, as reviewed by Planet et al. [48]. Our findings support this contention and advocate for whole-genome surveillance of other non-*S. aureus* populations in animals, which could lead to more accurate predictions of antibiotic resistance and the virulence of emergent clones. Ultimately, this can provide a better understanding of the enigmatic biological aspects that determine the recurrent strain dominance in endemic areas. In our investigation, we sequenced the genome of *Staphylococcus xylosus*, a coagulase-negative *Staphylococcus* that is often missed in conventional laboratory exams. *Staphylococcus xylosus* NM36’s unique virulence traits are a new variable in the complex epidemiology of mastitis in Basrah governorate.

## Conclusion

This research represents the first investigation into the genomic characteristics of *S. xylosus* within the geographical context of Iraq. This observation further underscores the need of using whole genome sequencing and comparative genomics analysis in order to get deeper insights into the origins and testing methodologies of multidrug-resistant isolates. Furthermore, there is a need to reassess microbiological and therapeutic approaches in the management of coagulase-negative staphylococci, especially in the context related to animal illnesses and public health.

### Supplementary Information

**Additional file 1. **

## Data Availability

The genome sequence of *Staphylococcus xylosus* NM36 has been deposited at GenBank—DDBJ/ENA/GenBank under the accession number GenBank: JARUHN000000000.1, GenBank assembly accession: GCA_029667155.1. The genome is associated with BioProject PRJNA950481 and BioSample SAMN33999823. The annotation was added by the NCBI Prokaryotic Genome Annotation Pipeline (PGAP https://www.ncbi.nlm.nih.gov/genome/annotation_prok/).

## Abbreviations

SCC*mec*: Staphylococcal cassette chromosome *mec*
CoNS: Coagulase-negative staphylococci
AMR: Antimicrobial resistant
NCBI: The National Center for Biotechnology Information
SNP: Single-nucleotide polymorphism
INDEL: Insertion/deletion
MDR: Multidrug resistant
MFS: The major facilitator superfamily
Contig: (From contiguous) overlapping DNA segments that together represent a consensus region of DNA
PATRIC: PathoSystems Resource Integration Center
RAST: Rapid Annotation using Subsystem Technology
PFGE: Pulse-field gel electrophoresis
MLST: Multilocus-sequence typing
PGAP: NCBI Prokaryotic Genome Annotation Pipeline
QC: Quality control
TV: NCBI Tree Viewer
CDS: Coding DNA sequence
MUSCLE: Multiple sequence comparison by log expectation
MSSA: Methicillin-sensitive *S. aureus*
SBS: Illumina sequencing by synthesis technology (Illumina HiSeq)

## References

[1] Akhaddar A, Elouennass M, Naama O, Boucetta M (2010). Staphylococcus xylosus isolated from an otogenic brain abscess in an adolescent. Surg Infect (Larchmt).

[2] Al-Mathkhury HJF (2008). Pathological study on Staphylococcus xylosus isolated from patients with urinary tract infections. J Al-Nahrain Univ Sci..

[3] Andrews S (2010) Babraham Bioinformatics - FastQC a quality control tool for high throughput sequence data. https://www.bioinformatics.babraham.ac.uk/projects/fastqc/. Accessed 22 Oct 2021

[4] Asante J, Amoako DG, Abia ALK, Somboro AM, Govinden U, Bester LA, Essack SY (2020). Review of clinically and epidemiologically relevant coagulase-negative staphylococci in Africa. Microb Drug Resist.

[5] Aziz RK, Bartels D, Best A, DeJongh M, Disz T, Edwards RA, Formsma K, Gerdes S, Glass EM, Kubal M, Meyer F, Olsen GJ, Olson R, Osterman AL, Overbeek RA, McNeil LK, Paarmann D, Paczian T, Parrello B, Pusch GD, Reich C, Stevens R, Vassieva O, Vonstein V, Wilke A, Zagnitko O (2008). The RAST server: rapid annotations using subsystems technology. BMC Genomics.

[6] Bankevich A, Nurk S, Antipov D, Gurevich AA, Dvorkin M, Kulikov AS, Lesin VM, Nikolenko SI, Pham S, Prjibelski AD, Pyshkin AV, Sirotkin AV, Vyahhi N, Tesler G, Alekseyev MA, Pevzner PA (2012). SPAdes: a new genome assembly algorithm and its applications to single-cell sequencing. J Comput Biol.

[7] Beenken KE, Blevins JS, Smeltzer MS (2003). Mutation of sarA in Staphylococcus aureus limits biofilm formation. Infect Immun.

[8] Boles BR, Horswill AR (2008). agr-mediated dispersal of Staphylococcus aureus biofilms. PLoS Pathog.

[9] Bose JL, Lehman MK, Fey PD, Bayles KW (2012). Contribution of the Staphylococcus aureus Atl AM and GL murein hydrolase activities in cell division, autolysis, and biofilm formation. PLoS ONE.

[10] Brandenberger M, Tschierske M, Giachino P, Wada A, Berger-Bächi B (2000). Inactivation of a novel three-cistronic operon tcaR-tcaA-tcaB increases teicoplanin resistance in Staphylococcus aureus. Biochim Biophys Acta - Gen Subj.

[11] Brettin T, Davis JJ, Disz T, Edwards RA, Gerdes S, Olsen GJ, Olson R, Overbeek R, Parrello B, Pusch GD, Shukla M, Thomason JA, Stevens R, Vonstein V, Wattam AR, Xia F (2015). RASTtk: a modular and extensible implementation of the RAST algorithm for building custom annotation pipelines and annotating batches of genomes. Sci Rep.

[12] Capra EJ, Laub MT (2012). Evolution of two-component signal transduction systems. Annu Rev Microbiol.

[13] Carrillo R, Téllez MDLÁ, Salinas S (2000). Staphylococcus xylosus: una bacteria emergente. Rev Medica Del Hosp Gen Mex.

[14] Chen M, Li Y, Li S, Cui W, Zhou Y, Qu Q, Che R, Li L, Yuan S, Liu X (2022). Molecular mechanism of Staphylococcus xylosus resistance against tylosin and florfenicol. Infect Drug Resist.

[15] Cheng Q, Xie G, Daligault H, Davenport K, Gleasner C, Jacobs L, Kubicek-Sutherland J, LeCuyer T, Otieno V, Raballah E, Doggett N, McMahon B, Perkins DJ, Mukundan H (2019). Genome sequence of a Staphylococcus xylosus Clinical isolate, strain SMA0341-04 (UGA5), from Siaya County Referral Hospital in Siaya, Kenya. Microbiol Resour Announc.

[16] Danecek P, Bonfield JK, Liddle J, Marshall J, Ohan V, Pollard MO, Whitwham A, Keane T, McCarthy SA, Davies RM (2021). Twelve years of SAMtools and BCFtools. Gigascience.

[17] Dashtbani-Roozbehani A, Brown MH (2021) Efflux pump mediated antimicrobial resistance by staphylococci in health-related environments: challenges and the quest for inhibition. Antibiotics 10. 10.3390/antibiotics10121502 10.3390/antibiotics10121502 PMC8698293 34943714

[18] Deng X, Sun F, Ji Q, Liang H, Missiakas D, Lan L, He C (2012). Expression of multidrug resistance efflux pump gene norA is iron responsive in Staphylococcus aureus. J Bacteriol.

[19] Dordet-Frisoni E, Talon R, Leroy S (2007). Physical and genetic map of the Staphylococcus xylosus C2a chromosome. FEMS Microbiol Lett.

[20] Ewing B, Hillier L, Wendl MC, Green P (1998). Base-calling of automated sequencer traces using Phred. I Accuracy Assessment Genome Res.

[21] Fijałkowski K, Struk M, Karakulska J, Paszkowska A, Giedrys-Kalemba S, Masiuk H, Czernomysy-Furowicz D, Nawrotek P (2014). Comparative analysis of superantigen genes in Staphylococcus xylosus and Staphylococcus aureus isolates collected from a single mammary quarter of cows with mastitis. J Microbiol.

[22] Frank JA, Reich CI, Sharma S, Weisbaum JS, Wilson BA, Olsen GJ (2008). Critical evaluation of two primers commonly used for amplification of bacterial 16S rRNA genes. Appl Environ Microbiol.

[23] Fujimura T, Murakami K (2008). Staphylococcus aureus clinical isolate with high-level methicillin resistance with an lytH mutation caused by IS1182 insertion. Antimicrob Agents Chemother.

[24] Gallegos MT, Schleif R, Bairoch A, Hofmann K, Ramos JL (1997). Arac/XylS family of transcriptional regulators. Microbiol Mol Biol Rev.

[25] Grant JR, Enns E, Marinier E, Mandal A, Herman EK, Chen C, Graham M, Van Domselaar G, Stothard P (2023) Proksee: in-depth characterization and visualization of bacterial genomes. Nucleic Acids Res 1–9. 10.1093/nar/gkad326 10.1093/nar/gkad326 PMC10320063 37140037

[26] Gustafson J, Strassle A, Hachler H, Kayser FH, Berger-Bachi B (1994). The femC locus of Staphylococcus aureus required for methicillin resistance includes the glutamine synthetase operon. J Bacteriol.

[27] Hong,  (2018). Complete genome sequence of biofilm-producing strain Staphylococcus xylosus S170. Korean J Microbiol.

[28] Jefferson KK, Pier DB, Goldmann DA, Pier GB, Al JET, Acteriol JB (2004) The teicoplanin-associated locus regulator (TcaR) and the intercellular adhesin locus regulator (IcaR) are transcriptional inhibitors of the ica locus in Staphylococcus aureus. 186:2449–2456. 10.1128/JB.186.8.2449 10.1128/JB.186.8.2449-2456.2004 PMC412131 15060048

[29] Kaur G, Arora A, Sathyabama S, Mubin N, Verma S, Mayilraj S, Agrewala JN (2016). Genome sequencing, assembly, annotation and analysis of Staphylococcus xylosus strain DMB3-Bh1 reveals genes responsible for pathogenicity. Gut Pathog.

[30] Kondo N, Kuwahara-Arai K, Kuroda-Murakami H, Tateda-Suzuki E, Hiramatsu K (2001). Eagle-type methicillin resistance: new phenotype of high methicillin resistance under MEC regulator gene control. Antimicrob Agents Chemother.

[31] Labrie SJ, El Haddad L, Tremblay DM, Plante PL, Wasserscheid J, Dumaresq J, Dewar K, Corbeil J, Moineau S (2014). First complete genome sequence of Staphylococcus xylosus, a meat starter culture and a host to propagate Staphylococcus aureus phages. Genome Announc.

[32] Lauderdale KJ, Boles BR, Cheung AL, Horswill AR (2009). Interconnections between Sigma B, agr, and proteolytic activity in Staphylococcus aureus biofilm maturation. Infect Immun.

[33] Leroy S, Christieans S, Talon R (2019). Tetracycline gene transfer in staphylococcus xylosus in situ during sausage fermentation. Front Microbiol.

[34] Leroy S, Vermassen A, Ras G, Talon R (2017) Insight into the genome of Staphylococcus xylosus, a ubiquitous species well adapted to meat products. Microorganisms 5. 10.3390/microorganisms5030052 10.3390/microorganisms5030052 PMC5620643 28850086

[35] Li H, Durbin R (2009). Fast and accurate short read alignment with Burrows-Wheeler transform. Bioinformatics.

[36] Ma APY, Jiang J, Tun HM, Mauroo NF, Yuen CS, Leung FCC (2014). Complete genome sequence of Staphylococcus xylosus HKUOPL8, a potential opportunistic pathogen of mammals. Genome Announc.

[37] Macfadyen AC, Harrison EM, Ellington MJ, Parkhill J, Holmes MA, Paterson GK (2018). A highly conserved mecC -encoding SCC mec type XI in a bovine isolate of methicillin-resistant Staphylococcus xylosus. J Antimicrob Chemother.

[38] Marchler-Bauer A, Bo Y, Han L, He J, Lanczycki CJ, Lu S, Chitsaz F, Derbyshire MK, Geer RC, Gonzales NR, Gwadz M, Hurwitz DI, Lu F, Marchler GH, Song JS, Thanki N, Wang Z, Yamashita RA, Zhang D, Zheng C, Geer LY, Bryant SH (2017). CDD/SPARCLE: functional classification of proteins via subfamily domain architectures. Nucleic Acids Res.

[39] Marchler-Bauer A, Bryant SH (2004). CD-Search: protein domain annotations on the fly. Nucleic Acids Res.

[40] Marchler-Bauer A, Derbyshire MK, Gonzales NR, Lu S, Chitsaz F, Geer LY, Geer RC, He J, Gwadz M, Hurwitz DI, Lanczycki CJ, Lu F, Marchler GH, Song JS, Thanki N, Wang Z, Yamashita RA, Zhang D, Zheng C, Bryant SH (2015). CDD: NCBI’s conserved domain database. Nucleic Acids Res.

[41] Marchler-Bauer A, Lu S, Anderson JB, Chitsaz F, Derbyshire MK, DeWeese-Scott C, Fong JH, Geer LY, Geer RC, Gonzales NR, Gwadz M, Hurwitz DI, Jackson JD, Ke Z, Lanczycki CJ, Lu F, Marchler GH, Mullokandov M, Omelchenko MV, Robertson CL, Song JS, Thanki N, Yamashita RA, Zhang D, Zhang N, Zheng C, Bryant SH (2011). CDD: a conserved domain database for the functional annotation of proteins. Nucleic Acids Res.

[42] Mashruwala AA, Gries CM, Scherr TD, Kielian T, Boyd JM (2017). SaeRS is responsive to cellular respiratory status and regulates fermentative biofilm formation in Staphylococcus aureus. Infect Immun.

[43] Mashruwala AA, Guchte A van de, Boyd JM (2017b) Impaired respiration elicits SrrAB-dependent programmed cell lysis and biofilm formation in Staphylococcus aureus. Elife 6. 10.7554/eLife.23845 10.7554/eLife.23845 PMC5380435 28221135

[44] Nikaido H, Takatsuka Y (2009). Mechanisms of RND multidrug efflux pumps. Biochim Biophys Acta - Proteins Proteomics.

[45] Overbeek R, Olson R, Pusch GD, Olsen GJ, Davis JJ, Disz T, Edwards RA, Gerdes S, Parrello B, Shukla M, Vonstein V, Wattam AR, Xia F, Stevens R (2014). The SEED and the Rapid Annotation of microbial genomes using Subsystems Technology (RAST). Nucleic Acids Res.

[46] Pasqua M, Bonaccorsi di Patti MC, Fanelli G, Utsumi R, Eguchi Y, Trirocco R, Prosseda G, Grossi M, Colonna B (2021). Host - bacterial pathogen communication: the wily role of the multidrug efflux pumps of the MFS family. Front Mol Biosci.

[47] Picard toolkit (2019) Broad Institute, GitHub repository. http://broadinstitute.github.io/picard/. Accessed 11 Apr 2022.

[48] Planet PJ, Narechania A, Chen L, Mathema B, Boundy S, Archer G, Kreiswirth B (2016). Architecture of a species: phylogenomics of Staphylococcus aureus. Trends Microbiol.

[49] Pohl K, Francois P, Stenz L, Schlink F, Geiger T, Herbert S, Goerke C, Schrenzel J, Wolz C (2009). CodY in Staphylococcus aureus: a regulatory link between metabolism and virulence gene expression. J Bacteriol.

[50] Rankin DJ, Rocha EPC, Brown SP (2011). What traits are carried on mobile genetic elements, and why. Heredity (Edinb).

[51] Saadoon AS (2022). Clinical and subclinical mastitis in buffalue in Mosul area, Iraq. Iraqi J Vet Sci.

[52] Savino S, Desmet T, Franceus J (2022). Insertions and deletions in protein evolution and engineering. Biotechnol Adv.

[53] Sayers EW, Beck J, Brister JR, Bolton EE, Canese K, Comeau DC, Funk K, Ketter A, Kim S, Kimchi A, Kitts PA, Kuznetsov A, Lathrop S, Lu Z, McGarvey K, Madden TL, Murphy TD, O’Leary N, Phan L, Schneider VA, Thibaud-Nissen F, Trawick BW, Pruitt KD, Ostell J (2020). Database resources of the National Center for Biotechnology Information. Nucleic Acids Res.

[54] Schilcher K, Horswill AR (2020). Staphylococcal biofilm development: structure, regulation, and treatment strategies. Microbiol Mol Biol Rev.

[55] Sharma-Kuinkel BK, Mann EE, Ahn JS, Kuechenmeister LJ, Dunman PM, Bayles KW (2009). The Staphylococcus aureus LytSR two-component regulatory system affects biofilm formation. J Bacteriol.

[56] Stothard P, Grant JR, Van Domselaar G (2018). Visualizing and comparing circular genomes using the CGView family of tools. Brief Bioinform.

[57] Tatusova T, Dicuccio M, Badretdin A, Chetvernin V, Nawrocki EP, Zaslavsky L, Lomsadze A, Pruitt KD, Borodovsky M, Ostell J (2016) NCBI prokaryotic genome annotation pipeline. Nucleic Acids Res 44. 10.1093/nar/gkw569 10.1093/nar/gkw569 PMC5001611 27342282

[58] Thompson MK, Keithly ME, Goodman MC, Hammer ND, Cook PD, Jagessar KL, Harp J, Skaar EP, Armstrong RN (2014). Structure and function of the genomically encoded fosfomycin resistance enzyme, FosB, from Staphylococcus aureus. Biochemistry.

[59] Tiwari N, López-Redondo M, Miguel-Romero L, Kulhankova K, Cahill MP, Tran PM, Kinney KJ, Kilgore SH, Al-Tameemi H, Herfst CA, Tuffs SW, Kirby JR, Boyd JM, McCormick JK, Salgado-Pabón W, Marina A, Schlievert PM, Fuentes EJ (2020) The SrrAB two-component system regulates Staphylococcus aureus pathogenicity through redox sensitive cysteines. Proc Natl Acad Sci U S A 117. 10.1073/pnas.1922523117 10.1073/pnas.1921307117 PMC7245129 32354997

[60] Toledo-Arana A, Merino N, Vergara-Irigaray M, Débarbouillé M, Penadés JR, Lasa I (2005). Staphylococcus aureus develops an alternative, ica-independent biofilm in the absence of the arlRS two-component system. J Bacteriol.

[61] Trotonda MP, Tamber S, Memmi G, Cheung AL (2008). MgrA represses biofilm formation in Staphylococcus aureus. Infect Immun.

[62] Vermassen A, de la Foye A, Loux V, Talon R, Leroy S (2014). Transcriptomic analysis of Staphylococcus xylosus in the presence of nitrate and nitrite in meat reveals its response to nitrosative stress. Front Microbiol.

[63] Wattam AR, Davis JJ, Assaf R, Boisvert S, Brettin T, Bun C, Conrad N, Dietrich EM, Disz T, Gabbard JL, Gerdes S, Henry CS, Kenyon RW, Machi D, Mao C, Nordberg EK, Olsen GJ, Murphy-Olson DE, Olson R, Overbeek R, Parrello B, Pusch GD, Shukla M, Vonstein V, Warren A, Xia F, Yoo H, Stevens RL (2017). Improvements to PATRIC, the all-bacterial bioinformatics database and analysis resource center. Nucleic Acids Res.

[64] Zarazaga M, Gómez P, Ceballos S, Torres C (2018) Molecular epidemiology of Staphylococcus aureus lineages in the animal–human interface. In: Fetsch A, editors. Staphylococcus aureus. Elsevier, Academic Press. pp 189–214. 10.1016/B978-0-12-809671-0.00010-3

[65] Zhao Y, Verma V, Belcheva A, Singh A, Fridman M, Golemi-Kotra D (2012). Staphylococcus aureus methicillin-resistance factor fmtA is regulated by the global regulator SarA. PLoS ONE.

